# *Brassica*-Derived Plant Bioactives as Modulators of Chemopreventive and Inflammatory Signaling Pathways

**DOI:** 10.3390/ijms18091890

**Published:** 2017-09-01

**Authors:** Christine Sturm, Anika E. Wagner

**Affiliations:** Institute of Nutritional Medicine, University of Lübeck, Ratzeburger Allee 160, 23538 Lübeck, Germany; christine.schuemann@gmail.com

**Keywords:** *Brassicaceae*, isothiocyanates, sulforaphane, Nrf2, NFκB, epigenetics

## Abstract

A high consumption of vegetables belonging to the *Brassicaceae *family has been related to a lower incidence of chronic diseases including different kinds of cancer. These beneficial effects of, e.g., broccoli, cabbage or rocket (arugula) intake have been mainly dedicated to the sulfur-containing glucosinolates (GLSs)—secondary plant compounds nearly exclusively present in *Brassicaceae—*and in particular to their bioactive breakdown products including isothiocyanates (ITCs). Overall, the current literature indicate that selected *Brassica*-derived ITCs exhibit health-promoting effects in vitro, as well as in laboratory mice in vivo. Some studies suggest anti-carcinogenic and anti-inflammatory properties for ITCs which may be communicated through an activation of the redox-sensitive transcription factor nuclear factor erythroid 2–related factor 2 (Nrf2) that controls the expression of antioxidant and phase II enzymes. Furthermore, it has been shown that ITCs are able to significantly ameliorate a severe inflammatory phenotype in colitic mice in vivo. As there are studies available suggesting an epigenetic mode of action for *Brassica*-derived phytochemicals, the conduction of further studies would be recommendable to investigate if the beneficial effects of these compounds also persist during an irregular consumption pattern.

## 1. Introduction

Chronic inflammatory diseases are a major risk factor for cancer development, especially regarding the gastro-intestinal tract [1,2]. In addition to the beneficial effect of inflammation as the primary response to infection and injury, a sustained production of inflammatory mediators, like cytokines and reactive oxygen species (ROS), may cause alterations in DNA integrity and lead to malignant cell transformation and cancer [3,4]. Cancer is a major public health problem and the incidence and mortality is still growing, currently accounting for over 12% deaths worldwide [5]. The concept of cancer chemoprevention was originally introduced by Wattenberg [6] and describes the protective mechanisms of natural or synthetic compounds that block the initiation of carcinogenesis. The identification of dietary compounds that may exert anti-inflammatory and chemopreventive actions and the investigation of the underlying cellular mechanisms is an important future challenge for improving cancer prevention. Especially, a high consumption of cruciferous vegetables, like cabbage, broccoli, and rocket (arugula), is associated with beneficial effects on the development of cancer [7,8,9]. Although the underlying mechanisms are yet not fully understood, the health-promoting effects of a diet rich in cruciferous vegetables have been linked to the breakdown products of glucosinolates (GLSs) [10,11,12,13].

## 2. Isothiocyanates (ITCs), the Bioactive Breakdown Products of Sulfur-Containing Glucosinolates (GLSs), Are Released by Myrosinase (MYR) Activity

**Figure 1:**
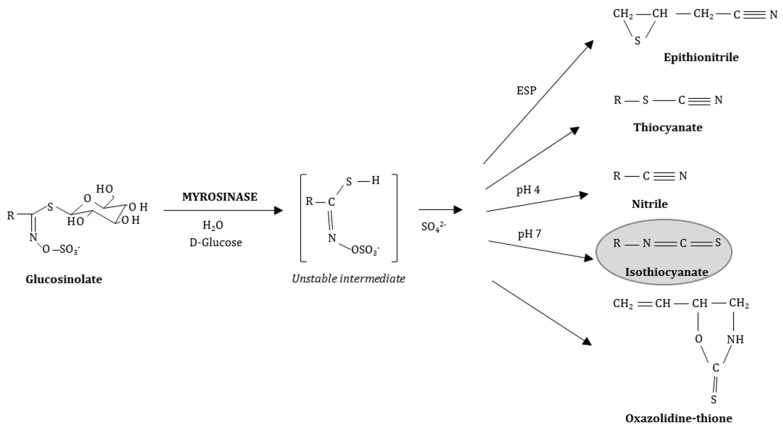
General structure of sulfur-containing glucosinolates (GLSs) and their breakdown products after hydrolysis by myrosinase (MYR) dependent of the reaction conditions [18]. ESP: epithiospecifier protein.

GLSs are characteristic secondary plant compounds present in *Cruciferae/Brassicaceae *and they are stable water-soluble *N*-hydroxysulfates with a sulfur-linked β-d-glucopyranose moiety and a variable amino acid-derived side chain (R) (Figure 1) [14]. Bioactive ITCs arise from their parent GLSs when the plant tissue is damaged by insect herbivores, during food preparation or chewing. Subsequently, the plant enzyme MYR, a thioglucohydrolase (E.C. 3.2.1.147), located in so-called myrosin cells separated from the GLSs, comes in contact with its GLS substrate previously stored apart in the vacuoles and catalyses the hydrolysis of the thioglucosidic bond [15,16,17]. Glucose is cleaved and the unstable aglycone thiohydroxamate-*O*-sulfonate spontaneously rearranges in various products depending on the specific parent GLS, the reaction pH and the presence of ferrous ions or the epithiospecifier protein (ESP) (Figure 1) [16].

In addition to plant-derived MYR-dependent GLSs cleavage, microorganisms within the murine and human gut seem to be capable of metabolizing GLSs to ITCs [19,20]. Table 1 gives an overview of the side chain chemical structures of selected GLSs. At neutral pH conditions, ITCs emerge from GLSs with aliphatic or aromatic side chains. The GLS glucoerucin (GER) is present in significant amounts in rocket seeds and sprouts and it is the precursor of 4-(methylthio) butyl-ITC (erucin) [21,22]. The GLSs glucoraphanin (GRA) and sinigrin (SIN), e.g., present in broccoli and red cabbage, yield sulforaphane (SFN; 4-(methylsulfinyl) butyl-ITC) and allyl-ITC (AITC; 2-propenyl-ITC), respectively. In vivo, erucin can be inter-converted to SFN [23,24,25,26]—the ITC that is the most extensively studied for its chemopreventive and anti-inflammatory properties in vitro, as well as in vivo [27,28,29].

## 3. Chemoprevention and Nuclear Factor Erythroid 2–Related Factor 2 (Nrf2)

The multi-stage process of carcinogenesis is divided into three operationally-defined stages: tumour initiation, then promotion, followed by tumour progression [32]. Endogenous repair mechanisms to maintain the integrity of genomic DNA can be impaired by oxidative stress induced by aerobic metabolism, the generation of ROS and reactive nitrogen species due to inflammation, ultraviolet radiation, pollutants, or dietary chemicals [33]. Initially, the exposure to endogenous or exogenous carcinogens may lead to irreversible genomic DNA damage. During this chemically-induced tumour initiation, the carcinogen interacts with nucleic acids leading to the activation of oncogenes and/or inactivation of tumour suppressor genes. The second step of carcinogenesis, tumour promotion, is a reversible process characterized by the expansion of initiated tumour cells and requires a sustained exposure to the carcinogen. Finally, the growth of a tumour with invasive and metastatic characteristics leads to the progression of neoplastic transformed cells [32]. Phytochemicals may regularly intervene in different stages of this process [34]. Blocking agents prevent carcinogens (1) from reaching target sites by inactivation or direct antioxidant activity, (2) from undergoing metabolic activation by inducing antioxidant enzymes, or (3) from interacting with crucial cellular macromolecules, even by epigenetic modifications [35,36]. Furthermore, chemoprevention may target progressed stages of carcinogenesis through the inhibition of proliferation and angiogenesis as well as through the induction of apoptosis and the attenuation of inflammation [37,38].

**Figure 2:**
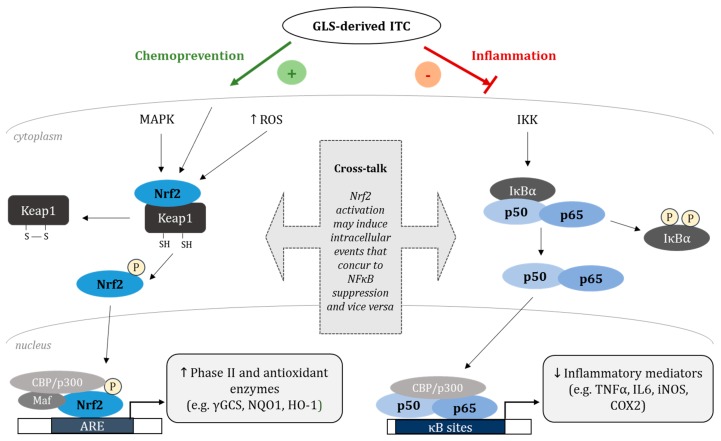
Proposed mechanisms of chemopreventive effects of GLS-derived isothiocyanates (ITCs) via nuclear factor erythroid 2–related factor 2 (Nrf2)-dependent antioxidant- and nuclear factor κB (NFκB)-mediated anti-inflammatory signalling pathways, considering a possible cross-talk between the two redox-sensitive transcription factors (modified from [18]). γGCS: γ-glutamylcysteine synthetase; NQO1: NAD(P)H quinone oxidoreductase 1, HO-1: heme oxygenase 1; TNFα: tumour necrosis factor alpha; IL6: interleukin 6; iNOS: inducible nitric oxide synthase; COX2: cyclooxygenase 2; IKK: IκB kinases; Keap1: Kelch-like ECH-associated protein 1; Maf: musculo aponeurotic fibrosarcoma; MAPK: mitogen-activated protein kinase; ROS: reactive oxygen species; ARE: antioxidant responsive element.

The induction of cytoprotective enzymes by phytochemicals is a crucial preventive step at the initiation stage of carcinogenesis and may protect DNA from damage and mutations. Previous studies show that GLS-derived compounds like ITC, and mainly SFN, are potent activators of the redox-sensitive transcription factor nuclear factor erythroid 2-related factor 2 (Nrf2) [39,40,41] which is a member of the cap ‘n’ collar family of basic leucine zipper transcription factors [42] and represents an important regulator of a battery of genes involved in chemoprevention and inflammation [43]. Under basal conditions, Nrf2 is bound to its inhibitor protein Kelch-like ECH-associated protein 1 (Keap1) leading to constant ubiquitination and degradation by the proteasome through Cullin 3-dependent E3 ubiquitin ligase [44]. Upon activation by electrophilic agents or oxidative stress, the cytosolic Keap1-Nrf2-complex is destroyed through the modification of cysteine residues of Keap1 [45], Nrf2 is released and translocates to the nucleus (Figure 2) [46]. The activation of Nrf2 can as well be mediated by mitogen-activated protein kinases (MAPKs), such as extracellular signal-regulated kinase (ERK), p38 or c-Jun NH_2_-terminal kinase (JNK), that transmit various extracellular signals into intracellular responses through serial phosphorylation cascades [47,48]. In the nucleus, Nrf2 dimerizes with small musculo aponeurotic fibrosarcoma (Maf) proteins and binds to the *cis*-acting antioxidant responsive element (ARE) located in the promotor region of target genes. Hence, Nrf2 induces the expression of genes including phase II and antioxidant enzymes like heme oxygenase 1 (HO-1), γ-glutamylcysteine synthetase (γGCS) and NAD(P)H quinone oxidoreductase 1 (NQO1) [49,50,51,52]. Phase II enzymes convert xenobiotics and potential carcinogens to inactive metabolites which can be readily excreted [53]. NQO1 is an obligate two-electron reductase that generates antioxidant forms of ubiquinone and vitamin E relevant for cytoprotection [54]. γGCS catalyzes the rate-limiting step in the biosynthesis of the intracellular antioxidant tripeptide glutathione (GSH) [55,56] and is composed of two subunits, the light chain (28 kDa) and the heavy chain (73 kDa), the latter possessing all the catalytic activity [57]. The antioxidant enzyme HO-1, the inducible form of HO, is ubiquitously expressed in systemic tissues and catalyses the initial and rate-limiting step in the degradation of heme into biliverdin/bilirubin, carbon monoxide (CO), and ferritin induced by the release of free iron [58]. The metabolic products of HO-1 reaction have distinct anti-oxidative and anti-inflammatory functions important for the cellular homeostasis in response to ROS-mediated stress [59,60,61,62,63,64,65,66] and therefore the induction of HO-1 gene expression may be a compromising target in cancer chemoprevention.

## 4. Nuclear Factor κB (NFκB) in Inflammation and Cancer

The transcription factor NFκB regulates the expression of genes involved in multiple biological processes including inflammation, cell proliferation and apoptosis [67] and might be a key link between inflammation and cancer [68]. “NFκB” is used as a generic term for a protein family including five main proteins, namely RelA (p65), RelB, c-Rel, NFκB1 (p50/p105), and NFκB2 (p52/p100) occurring in various (combined) dimeric complexes [69]. In unstimulated resting cells, the dimeric transcription factor remains transcriptionally inactive in the cytoplasm bound to its specific inhibitory proteins (IκBs). Phosphorylation by IκB kinases (IKK), ubiquitination and proteasomal degradation of the IκBs lead to the release of NFκB from IκBs and, thereby, activation of NFκB. Activated NFκB translocates to the nucleus where it binds to specific DNA sequences in the promotor region of its target genes [67,70]. In the classic (canonical) NFκB transactivation pathway, the p50/p65 heterodimer is basically activated by pro-inflammatory cytokines like interleukin (IL) 1 beta (IL1β) and tumour necrosis factor alpha (TNFα) and by viral infections resulting in an enhanced expression of multiple inflammatory and innate immune genes (e.g., the cytokines IL6, IL1β, TNFα and the chemokine IL8). Moreover, expression of the inducible effector enzymes cyclooxygenase 2 (COX2) and inducible nitric oxide synthase (iNOS) and adhesion molecules (e.g., epithelial cell adhesion molecule (Epcam)) [71] is elevated. An alternative (non-canonical) pathway for NFκB activation is triggered by cytokines of the TNF family such as lymphotoxin β and B-cell activating factor (BAFF) and the activation of a subset of necessary receptors predominantly results in the nuclear translocation of p52–RelB dimers [72]. This alternative pathway seems to play a pivotal role regarding the expression of genes involved in the development of secondary lymphoid organs, such as the spleen and lymph nodes and adaptive immune response [73,74]. Regarding tumourigenesis, NFκB target genes are involved in all steps of tumour cell development comprising cell survival, proliferation, tumour invasion, and angiogenesis to final metastasis [2]. Even though the physiological function of this signalling cascade is the protection of the cell from harm, a deviating activation may provoke the transition from inflammation to cancerous growth. Hence, the inhibition of pro-inflammatory NFκB signalling pathways may be a critical mechanism in preventing inflammation-associated diseases.

## 5. Protection against Colitis by Targeting Nrf2

In addition to genetic, environmental, and intestinal microbial factors [75], a dysregulation regarding the luminal microflora and dietary antigens are discussed to contribute to the pathology of the chronic and relapsing inflammatory bowel diseases (IBDs) colitis ulcerosa (UC), and Crohn´s disease (CD) [76]. CD is characterized by transmural inflammation that may occur at any site of the gastrointestinal tract from mouth to anus, but mainly affects the terminal ileum and the colon, whereas UC strikes the large bowel and is manifested in mucosal and sub-mucosal inflammation and ulceration [76]. In IBD patients, the disruption of the epithelial cell barrier involves an errant balance of the immune homeostasis between tolerance to the intestinal host microflora, on the one hand, and initiation of inflammation by the secretion of cytokines, chemokines, and antimicrobial agents on the other hand [77]. Gastrointestinal-related symptoms of patients suffering from IBD are diarrhoea, abdominal pain and cramping, permanent blood loss from chronically-inflamed mucosa, loss of appetite, weight loss, and micronutrient deficiencies [78,79]. The sustained chronic inflammation dramatically increases the risk for developing a colitis-associated colorectal cancer—the most serious long-term complication in IBDs [80]. The disease patterns of human IBDs can be experimentally mimicked in mice by the induction of a colitis development by the application of dextran-sodium sulphate (DSS) in the drinking water [81]. The regular rectal localization of DSS-induced colitis in mice is comparable to human UC, whereas histological alterations, like transmural inflammation, due to the DSS administration characterize CD [81,82]. DSS exhibits a direct toxicity to colonic epithelial cells [83] and causes erosions of the epithelium accompanied by an increase in permeability of the colonic mucosa for large molecules like DSS [84]. The assessment of clinical parameters, like weight loss and faecal occult blood during DSS administration in combination with histopathological analyses of the colon, allows the evaluation of the effect of secondary plant compounds or xenobiotics/drugs on intestinal inflammation using a therapeutic or even a preventive experimental approach [85,86,87].

In DSS-induced animal models of experimental colitis, the generation of ROS by epithelial cells and infiltrating inflammatory cells have been shown to be a driving force of tissue damage and reflects the underlying pathophysiology of UC [88,89,90]. Nrf2 regulates the transcription of several detoxification enzymes and antioxidant proteins involved in the cellular defence against oxidative stress [91]. Nrf2-deficient mice tend to be more susceptible to DSS-induced colitis in comparison with their wild type counterparts, which was shown to be associated with the reduction of phase II detoxifying/antioxidant enzymes and the elevation of inflammatory markers in Nrf2-null mice [92]. Additionally, a Nrf2 gene polymorphism that reduces the activity of Nrf2 has been shown to be associated with an increased risk of developing ulcerative colitis in a Japanese population [93].

Several dietary ingredients/plant compounds have been identified to possess the capacity to alleviate experimentally induced colitis via an Nrf2 pathway activation. Therefore, the decrease of oxidative stress as a target for the therapeutic treatment of IBD, with e.g., 5-aminosalicylic acid preparations like mesalazine [94], may be complemented by dietary interventions.

In our own study, pre-treating C57BL/6 mice with the ITC sulforaphane for seven days ameliorated a DSS-induced colitis phenotype which has been approved by an amendment of the disease activity index, the colon length, the appearance of the intestinal mucosa assessed by colonoscopy, the histopathology and the expression levels of pro-inflammatory markers and Nrf2-regulated cytoprotective enzymes [95]. Resistant glycogen reduced colitis in experimental mice models of colitis by decreasing oxidative stress and increasing HO-1 expression in the large intestine of mice. This effect was shown to be triggered by the activation of (ERK1/2)- and JNK signalling and subsequent phosphorylation of Nrf2 in macrophages [96]. Khodir and co-workers [97] investigated the coloprotective potential of coenzyme Q10 in a rat model of experimental colitis and documented the Nrf2/HO-1 pathway to be mainly responsible for the suppression of inflammatory marker release and the recovery of the oxidant/antioxidant homeostasis. Additionally, luteolin (3’, 4’, 5, 7-tetrahydroxyflavone) attenuated disease patterns of a DSS-induced colitis in mice and activated Nrf2-dependent gene expression of HO-1 and NQO1 [98].

In human NCM460 colonocytes, CPUY192018, an inhibitor of the Keap1-Nrf2 protein-protein interaction, activated the Nrf2 pathway resulting in an increase of both, cellular and nuclear Nrf2 protein levels which in consequence increased the expression of Nrf2 downstream cytoprotective genes [91]. Interestingly, the DSS-mediated induction of oxidative stress and cell damage in NCM460 cells was antagonized by the inhibitor CPUY192018. Furthermore, in a DSS-induced mouse model of UC, a CPUY192018 treatment alleviated disease symptoms accompanied by a decreased expression of inflammatory cytokines TNFα, IL6, and IL1β, and a significant up-regulation of Nrf2 and its cytoprotective target genes HO-1, γGCS, and GPx2 in the colon of CPUY192018-treated DSS-mice [91].

## 6. GLS-Derived Phytochemicals Modulate Inflammation by Inducing Nrf2 and Suppressing NFκB

So far, various ITCs have been identified as potent Nrf2 inducers. In our own studies, the ITCs AITC, 2-phenylethyl-isothiocyanate (PEITC), and butyl ITC induced phase II and antioxidant enzymes via Nrf2 in cultured murine fibroblasts which was attended by the activation of the upper MAPK ERK1/2 [39]. SFN is the most intensively-studied ITC and a prominent plant-derived Nrf2 inducer in vitro, as well as in vivo [41,52,99,100], which also shows anti-inflammatory activities [95,101,102]. Figure 2 gives an overview of postulated mechanisms by which ITCs activate Nrf2 and suppress NFκB and downstream target gene expressions. Overall, the underlying mechanisms of how ITCs modulate the NFκB pathway are poorly understood. The aberrant activation of NFκB and its target genes associated with the inhibition of apoptosis, induction of cell cycle progression, angiogenesis, and metastasis is a crucial link between inflammation and cancer [103]. Furthermore, NFκB is involved in physiological processes, such as the innate and adaptive immune responses regulating the transcription of inflammatory mediators, including cytokines, chemokines, proteases, and COX2 [104]. The modulation of the NFκB signalling pathway by GLS breakdown products has been shown to block pro-inflammatory signals in vitro and in vivo [105]. DIM has been described to reduce the nuclear translocation of the p65 NFκB subunit and its transcriptional activity by repressing IKK/IκB phosphorylation. This results in lower levels of inflammatory mediators such as iNOS and COX2 in LPS-activated macrophages [106], during brain inflammation [107], as well as during colonic inflammation in mice [108,109]. Anti-inflammatory/NFκB-inhibitory effects of both SFN and PEITC have been described in stimulated macrophages and cancer cell lines [110,111,112,113,114], as well as under acute and chronic DSS-induced inflammatory settings in the colon of mice [115]. PEITC and SFN have also been shown to reverse the UV-induced apoptosis in HaCaT keratinocytes and ex vivo skin samples [116]. Park and co-workers [117] revealed a suppressive effect of PEITC on LPS-stimulated toll-like receptor (TLR) signalling during inflammation. The lower levels of TLR adaptor molecules inhibited the activation of the transcription factors NFκB and interferon regulatory factor 3 and, consequently, the production of pro-inflammatory cytokines and type I interferons [117]. SFN may also decrease DNA binding of NFκB subunits without affecting the nuclear translocation [101]. A direct interaction of SFN with NFκB subunits was suggested via dithiocarbamate formation and binding to essential Cys residues of NFκB subunits [101]. On the other hand, the inhibition of NFκB DNA binding by SFN was suggested to be indirectly mediated by the negative modulation of the thioredoxin/thioredoxin reductase system responsible for regulating NFκB DNA binding [118]. The involvement of redox modulation and thiol reactivity in the regulation of NFκB-dependent transcription by SFN was substantiated by a study from Kim and colleagues [119], where SFN selectively inhibited NFκB activation through an interaction with thiol groups of NFκB in an in vitro model of osteoclastogenesis. Furthermore, SFN downregulated COX2 expression in LPS-activated murine macrophages by inhibiting transcriptional coactivators of NFκB, namely CCAAT/enhancer binding proteins (C/EBP), cAMP responsive element binding protein (CREB), and activator-protein 1 (AP-1), in the COX2 gene promotor [102].

ITCs may also directly inhibited the pro-inflammatory cytokine macrophage migration inhibitory factor (MIF) by covalently modifying the N-terminal proline residue of MIF, which resulted in the loss of catalytic tautomerase activity and disruption of protein conformation [120,121,122,123].

SFN seems to directly inactivate NFκB subunits or relevant co-activators in the nucleus by thiol-dependent modifications without interfering with the nuclear translocation of the p50 and p65 NFκB subunits [101]. COX2 expression induced by LPS-stimulation was suppressed in murine macrophages by SFN via the modulation of different promoter activities involved in the COX2 transcriptional regulation [102]. Furthermore, both PEITC and benzyl ITC reduced the secretion and mRNA level of pro-inflammatory cytokines IL6, IL1β, and TNFα in LPS-stimulated RAW 264.7 macrophages [124,125]. PEITC and benzyl ITC inhibited LPS-induced NFκB signalling by preventing the LPS-induced increase in phospho-IκBα levels and by the inhibition of p65 nuclear translocation as well as by the suppression of NFκB DNA binding activity.

## 7. The Nrf2-NFκB Cross-Talk

Sustained oxidative stress is a major cause of chronic inflammation and the development of cancer [104]. The Nrf2-dependent signalling pathway is crucial for the cellular defence against oxidative stress and a modulation of the Nrf2 pathway may also affect the redox-sensitive pro-inflammatory response regulated by the transcription factor NFκB [126]. Interestingly, the bioactivity of the majority of phytochemicals, including SFN and curcumin, was demonstrated to be mediated by anti-inflammatory as well as antioxidant properties [127,128,129,130]. A possible cross-talk of redox-sensitive NFκB- and Nrf2 signalling pathways may exist concerning the phosphorylation of the transcription factors as well as their upstream kinases and thiol modifications/oxidation of their inhibitor proteins resulting in a concurrent regulation of downstream gene expression levels [131].

Studies in Nrf2 knockout mice demonstrate an interplay between the Nrf2 and NFκB pathway because animals lacking Nrf2 showed an inflammatory phenotype (e.g., nephritis, brain injury, retinal disease, colitis) together with an augmentation of cytokine production compared with the wild-type animals [92,132,133,134,135].

Liu and colleagues [43] observed a suppression of the Nrf2-ARE signalling pathway by the NFκB subunit p65 in vitro, which was not triggered by a direct interaction of p65 with ARE-associated proteins such as Keap1, Nrf2 and small Maf protein MafK or an interference of p65 with protein stability, subcellular localization and DNA-binding activity of Nrf2. Actually, Nrf2 was inactivated by the deprivation of the coactivator CREB binding protein (CBP) and p65 promoted the recruitment of histone deacetylase (HDAC) 3, which is a co-repressor of ARE. The anti-oxidative and anti-inflammatory effects of ethyl pyruvate in a microglia cell line were found to be mediated by a nuclear accumulation of Nrf2 and the recruitment of p300, a transcriptional co-activator for both Nrf2 and p65, which inhibited an interaction of p65 with p300 and impeded the expression of LPS-induced iNOS expression [136].

In a murine colitis model, a synthesized chalcone derivative significantly ameliorated chemically-induced colonic damage and mucosal ulcerisation [137]. In HT-29 cells, a pre-treatment with the chalcone derivative inhibited the TNF-α-induced NFκB activation and activated Nrf2 via ERK1/2 and p38 and enhanced HO-1 expression. The authors suggest that the subsequent induction of HO-1 protein interferes with the nuclear translocation step of NFκB subunit p65 without affecting IκBα phosphorylation and degradation [137]. Bilirubin and CO are metabolites/products of the HO-1 catalyzed cleavage of the porphyrin ring of heme and additionally both display anti-inflammatory effects protecting against experimental colitis in mice [138,139,140,141,142]. The activation of Nrf2 and the induction of HO-1 expression by the flavonoid quercetin also repressed the expression of NFκB and pro-inflammatory markers in the livers of nickel-treated mice [143]. Moreover, in LPS-activated macrophages and septic mice, an induction of HO-1 inhibited the expression of pro-inflammatory mediators through Nrf2 activation and NFκB inhibition [144].

The loss of the coordinated balance between Nrf2 and NFκB pathways in handling the inflammatory response of a cell/tissue is associated with several diseases [145]. The cross-talk between the Nrf2 and NFκB pathway occurs through diverse complex molecular interactions including transcriptional and post-transcriptional mechanisms [146]. However, details of the co-regulation and negative feedback in this cross-talk are not yet fully elucidated. The consumption of Nrf2 activating and anti-inflammatory food-derived compounds may be a promising strategy in disease prevention by attenuating chronic inflammation.

## 8. Epigenetic Mechanisms: Relevance for the Nrf2 Pathway and Epigenetic Impact of *Brassica*-Derived Phytochemicals

Cellular organization of the genomic DNA is achieved via the chromatin structure and this defined compact structure greatly influences the ability to activate or silence genetic information. Epigenetics describes the study of heritable changes in gene expression that occur independently of changes in the primary DNA sequence [147]. Epigenetic modifications, namely DNA methylation, histone modifications and nucleosome positioning, are important regulators of cellular processes, including gene and microRNA (miRNA) expression, DNA-protein interactions, and cellular differentiation [148]. DNA methylation at the 5′ position of cytosine residues within CpG dinucleotides catalysed by DNA methyltransferases (DNMT: DNMT1, DNMT3a, and DNMT3b) epigenetically controls DNA stability and integrity and often occurs within regulatory regions of genes [149]. A hypermethylation of CpG islands usually silences gene expression, while demethylation often reactivates genes [149,150]. Histone acetyltransferases (HATs) transfer acetyl groups to lysine residues in histone tails resulting in an open chromatin structure and an activation of genes. On the other hand, HDACs remove histone acetyl groups by catalysing their transfer to coenzyme A and regulate gene silencing by preserving the condensed chromatin state [151].

Cancer cells show epigenetic characteristics like global DNA hypomethylation, altered cellular HDAC activity and modified miRNA expression [152]. An uncontrolled proliferation may be the consequence of epigenetic silencing of e.g., detoxifying enzymes, tumour suppressor genes, cell cycle regulators, and genes responsible for DNA repair or apoptosis. Due to the reversible nature of epigenetic aberrations, a modulation of epigenetically caused changes in gene expression by phytochemicals may be a promising approach in cancer prevention at the initiation step of carcinogenesis [153].

In vitro studies, using different cancer cells, have shown that, besides ITCs, bioactive food components including polyphenols, allyl compounds, folate, selenium, retinoids, and fatty acids influence global DNA hypomethylation, tumour suppressor gene promoter hypermethylation and histone modifications [154].

Wong and coworkers [155] investigated the genome-wide effects of SFN and DIM on promoter methylation in normal prostate epithelial cells and prostate cancer cells. Both SFN and DIM reversed many of the cancer-associated promotor methylations, including abnormally-methylated genes that are dysregulated during cancer progression (e.g., cell migration, cell adhesion, cell-cell signalling, and transcriptional regulation) [155]. In cultured mouse skin epidermal cells, SFN had an anti-cancer effect involving epigenetic modifications [156]. SFN inhibited the neoplastic transformation of the mouse skin, chemically induced by 12-*O*-tetradecanoylphorbol-13-acetate, by decreasing the methylation in the promotor region of the Nrf2 gene, resulting in an increased Nrf2 mRNA expression. Furthermore, SFN reduced the protein expression of DNMT1, DNMT3a, and DNMT3b. The total HDAC activity and the protein expression of HDAC1, HDAC2, HDAC3, and HDAC4 was also decreased by the treatment of the cells with 2.5 μM SFN for five days. Accordingly, Zhang and colleagues [157] observed an effect of 2.5 μM SFN on epigenetic mechanisms, including the demethylation of the Nrf2 promotor and the subsequent activation of the Nrf2 pathway in murine prostate cancer cells after five days. This effect of hypomethylation was associated with a decreased protein expression of DNMT1 and DNMT3a as well as reduced protein expression levels of HDAC1, HDAC4, HDAC5, and HDAC7. In addition to SFN, DIM also targeted Nrf2 via epigenetic modification and exerted chemopreventive effects in prostate cancer cells and in transgenic adenocarcinoma of mouse prostate (TRAMP) mice in vivo [158]. Like SFN [157], DIM (2.5 μM in vitro for five days and 1% DIM in the diet for 16 weeks in vivo) reduced the methylation of the first five CpGs in the promotor region of Nrf2. In TRAMP-C1 cells, the effect of DIM to demethylate the Nrf2 promotor correlated with its potential to decrease the expression of DNMT and HDAC. The development and promotion of human prostate cancer has been found to be associated with epigenetic alterations [159] and Nrf2 was shown to be epigenetically silenced in TRAMP mice [160]. Hence, the demethylation of the Nrf2 gene and the re-activation of the Nrf2 expression by *Brassica*-derived phytochemicals like SFN and DIM, but also by curcumin [161] or tocopherols [162], may be an important target for cancer chemoprevention.

In mice, the protection against intestinal carcinogenesis and the suppression of growth of human prostate cancer xenografts by SFN was accompanied with epigenetic histone modifications and was associated with an inhibition of HDAC activity [163,164]. Additionally, in human subjects, the consumption of SFN-rich broccoli sprouts resulted in the inhibition of HDAC activity [163].

miRNAs are endogenous small non-coding RNA molecules of 17–25 nucleotides in length that negatively interfere with gene expression by inhibiting the translation and/or triggering the degradation of target messenger RNAs and, hence, play an important role in epigenetic regulation of gene expression [165,166,167]. miRNAs may contribute to carcinogenesis by a multitude of mechanisms, such as by modulating apoptosis, angiogenesis or the expression of genes involved in cell migration/invasion [168]. There is evidence from cell culture studies, as well as from in vivo experiments, that *Brassica*-derived compounds, such as DIM, indole-3-carbinol (I3C), AITC, PEITC, butyl-ITC (BITC), and SFN, modulate the abnormal expression of miRNAs in different types of cancer. Hence, their chemopreventive effect may be partly mediated through their function as potent miRNA regulators [40,169,170,171,172,173,174,175,176].

For example, PEITC and I3C attenuated the altered expression of several miRNAs in the lung of rats following the exposure to environmental cigarette smoke (ECS) [171]. PEITC counteracted the expression of ECS-downregulated miRNAs involved in cellular mechanisms, such as stress response, NFκB activation, cell proliferation, apoptosis, and angiogenesis. I3C additionally regulated miRNAs responsible for p53 function [171]. I3C positively modulated deregulated onco-miRNAs, such as miR-21 in lung tissues of mice exposed to the carcinogen vinyl carbamate [176]. Regarding the epithelial-to-mesenchymal transition of pancreatic cancer cells, DIM functioned as a modulator of miRNA expression. In detail, DIM up-regulated miR-200 and the let-7 family, which were increased in gemcitabine-resistant pancreatic cancer cells, leading to the reversal of the cells to an epithelial phenotype [177]. The induction of miR-let-7a expression has been shown to be mediated by SFN and was involved in the inhibition of K-ras expression and cancer stem cell characteristics during pancreatic ductal adenocarcinoma progression [169].

In recent years, several miRNAs have been identified to modulate the Nrf2/Keap1 signalling pathway epigenetically. Dysregulated miRNA expression during tumour progression may result in elevated Nrf2 activity and tumour growth/cell survival due to the downregulation of affector miRNAs that normally decrease Nrf2 mRNA level [178]. On the other hand, some effector miRNAs are influenced by the gain of Nrf2 activity which in consequence causes chemoresistance and, hence, an alleviation of therapeutic success [178]. As such affector miRNAs, that act independently from the interaction of Nrf2 with Keap1, miRNAs miR-153, miR-27-a, miR-142-5p, and miR-144 regulated the Nrf2 expression in neuroblastoma cells [179], and miR-28 targeted the 3’UTR of Nrf2 mRNA decreasing Nrf2 expression in human breast cancer cells [180]. Increased oxidative stress in sickle erythrocytes and intravascular haemolysis was shown to be associated with reduced expression levels Nrf2 and Nrf2-regulated genes [181,182]. In erythrocytes from patients affected by sickle cell disease, miR-144 expression was upregulated while a direct regulatory effect on Nrf2 expression and two putative binding sites for miR-144 in the 3’UTR of Nrf2 mRNA were identified [183]. An oncogenic potential and a regulatory effect of miR-93 on Nrf2 protein and Nrf2 target gene expression was observed in a rat model of mammary carcinogenesis [184]. Recently, Wasik and colleagues [185] found Nrf2 and its target gene expressions to be reduced in liver specimens from patients with primary biliary cholangitis, which was associated with an overexpression of miR-132 and miR-34a and increased protein levels of both Keap1 and p62.

On the other hand, an elevated expression of Nrf2 in specific tumour cell lines was shown to be associated with a downregulated expression of miR-1 and miR-206 [186] and the up- and downregulation of miR-125-b1 and miR-29-b1, respectively [187]. miRNAs may also affect Nrf2 activity by posttranscriptional regulation of Keap1. For example, miR-200-a and miR-141 were found to bind to the 3’-UTR of the Keap1 transcript leading to reduced Keap1 protein levels and an enhanced transcriptional activity of Nrf2 [188,189,190,191]. The Nrf2-dependent induction of miRNAs was shown to interact with other molecular pathways, as miR-125b is increased by Nrf2, and inhibited aryl hydrocarbon receptor (AhR) repressor, which contributed to the protection from acute kidney injury [192]. A study from our group revealed an interaction between the Nrf2 and the pro-inflammatory NFκB pathway that was affected by miR-155 [40]. The expression of miR-155 in murine macrophages was downregulated by AITC and was associated with Nrf2 activation and a significant reduction of pro-inflammatory TNFα expression.

## 9. Data from Clinical Trials on the Effects of ITCs In Vivo

During normal gastrointestinal passage, the intestinal cells are the first line of defence. Therefore, these cells come into direct contact with ITCs which are then absorbed by passive diffusion [193]. Following the absorption, ITCs are conjugated to GSH by glutathione-*S*-transferase (GST), cleaved by γ-glutamyltranspeptidase and dipeptidase and enter the circulation being initially transported to the liver. Thereafter, *N*-acetyltransferase forms *N*-acetylcysteine conjugates which are further processed to mercapturic acid which is transported to the kidney to be secreted via the urine [18,193,194]. The inactivation of the ITC-releasing enzyme MYR either by cooking fresh broccoli or by the blanching-freezing procedure performed for commercially-available frozen broccoli was shown to reduce the bioavailability of ITCs in humans [26,195]. Furthermore, two studies in human subjects showed that the bioavailability of SFN was significantly lower following the consumption of broccoli supplements or GRA-rich broccoli powder lacking MYR compared to fresh or air-dried broccoli sprouts [24,196]. Additionally, inter-individual variances in ITC metabolism and excretion may exist due to differences in GST-genotypes [197,198].

An induction of Nrf2-regulated cytoprotective genes was reported in healthy subjects after the topical application of SFN-rich broccoli sprout extract in human skin [199] and in nasal lavage cells following an oral administration of SFN [200]. In patients with chronic obstructive pulmonary disease (COPD), a treatment with SFN induced Nrf2 activity and cytoprotective enzymes in alveolar macrophages [201]. However, Wise and coworkers [202] did not observe an effect on Nrf2 and inflammatory markers by a four week supplementation of both, 25 and 150 μM SFN/day in alveolar macrophages and bronchial epithelial cells of COPD patients relative to baseline levels and compared to the placebo group. In a randomized controlled trial investigating an effect of cruciferous vegetable consumption on systemic inflammation in healthy subjects a significantly lower level of serum IL6 concentrations in response to a 14-day two-dose cruciferous vegetable diet (14 g/kg bodyweight) were detected, whereas no changes in other inflammatory biomarkers (e.g., IL8, C-reactive protein, TNFα) could be observed [203].

## 10. Conclusions

Chemoprevention by dietary ingredients displays an inexpensive, easily-applicable, and readily-accessible approach which may prevent the onset of chronic diseases, including cancer. Although research on the underlying molecular mechanisms by which *Brassica*-derived phytochemicals mediate their health-promoting effects has been conducted for some decades, the understanding of the signalling pathways involved still remains mostly unclear. In addition to an effect on chemopreventive and inflammatory pathways it has been documented that ITCs and other GLS breakdown products also target epigenetic mechanisms such as histone modifications and DNA methylation which may contribute to its health-promoting effects. However, further analyses, especially with regard to in vivo studies comprising animal models and human subjects, are needed to further elucidate the underlying mechanisms being responsible for the protective effects of *Brassica*-derived plant bioactives in the development of chronic diseases.

## Figures and Tables

**Table 1:** Food sources of the GLS test compounds and chemical structures of the GLS side chains.

Parent Glucosinolate	Side Chain	Isothiocyanate	Food Sources *
Glucoerucin (GER)	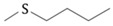	Erucin	Rocket
Gluoraphanin (GRA)	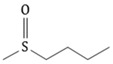	Sulforaphane (SFN)	Broccoli, red cabbage
Sinigrin (SIN)	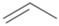	Allyl-isothiocyanate (AITC)	Brussels sprouts, red and white cabbage, kale
Gluconasturtiin (GSTI)	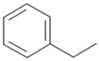	2-phenylethyl-isothiocyanate (PEITC)	Watercress, turnip, swede

* [21,30,31].
